# Serum Neurofilament Light Chain and GFAP Levels Are Associated with Structural Brain Connectivity in Parkinson’s Disease

**DOI:** 10.3390/ijms27093934

**Published:** 2026-04-28

**Authors:** Maria Celeste Bonacci, Jolanda Buonocore, Camilla Calomino, Maria Giovanna Bianco, Matteo Battocchio, Pietro Bontempi, Alessandro Daducci, Costanza Maria Cristiani, Aldo Quattrone, Maria Eugenia Caligiuri, Andrea Quattrone

**Affiliations:** 1 Department of Medical and Surgical Sciences, University Magna Graecia of Catanzaro, 88100 Catanzaro, Italy; mariaceleste.bonacci@unicz.it (M.C.B.); jolanda.buonocore@unicz.it (J.B.); camilla.calomino@unicz.it (C.C.); mg.bianco@unicz.it (M.G.B.); costanza.cristiani@unicz.it (C.M.C.); an.quattrone@unicz.it (A.Q.); 2 Neuroscience Research Center, University Magna Graecia of Catanzaro, 88100 Catanzaro, Italy; quattrone@unicz.it; 3 Institute of Neurology, Department of Medical and Surgical Sciences, University Magna Graecia of Catanzaro, 88100 Catanzaro, Italy; 4 Diffusion Imaging and Connectivity Estimation (DICE) Lab, Department of Computer Science, University of Verona, 37129 Verona, Italy; matteo.battocchio@univr.it (M.B.); alessandro.daducci@univr.it (A.D.); 5 Department of Engineering for Innovation Medicine, University of Verona, 37129 Verona, Italy; pietro.bontempi@univr.it

**Keywords:** Parkinson’s disease, diffusion-weighted tractography, graph-analysis, neurofilament light chain, glial fibrillary acidic protein

## Abstract

Parkinson’s disease (PD) is a progressive neurodegenerative disorder characterized by motor and non-motor symptoms and widespread alterations in brain networks. Circulating biomarkers such as neurofilament light chain (NfL) and glial fibrillary acidic protein (GFAP) reflect neuroaxonal damage and astroglial activation, respectively, but their relationship with large-scale brain connectivity remains poorly understood. Seventy-three PD patients and thirty-four healthy controls underwent diffusion magnetic resonance imaging. Whole-brain tractography was used to reconstruct structural connectivity networks, and graph-theoretical measures were derived. Serum NfL and GFAP levels were quantified, and their associations with network metrics and clinical variables were assessed. PD patients showed significant alterations in global and nodal network organization compared to controls. Higher NfL and GFAP levels were associated with reduced global clustering coefficient and efficiency, as well as increased path length and modularity. At the regional level, higher biomarker levels were associated with reduced network measures in the right thalamus and right cerebellar cortex. No significant associations were observed in healthy controls. These findings demonstrate that circulating biomarkers of neurodegeneration are linked to both global and regional disruptions of structural brain connectivity in PD, supporting the integration of blood-based biomarkers and connectomics to better characterize disease-related network alterations.

## 1. Introduction

Parkinson’s disease (PD) is a progressive neurodegenerative disorder characterized by a wide spectrum of motor and non-motor manifestations, including bradykinesia, rigidity, resting tremor, postural instability, gait impairment, cognitive deficits, and autonomic dysfunction [1]. PD is characterized by the accumulation of misfolded α-synuclein, progressive neuronal loss, and neuroinflammatory processes. There is growing interest in investigating these pathological mechanisms in vivo through accessible biomarkers measured in cerebrospinal fluid (CSF) as well as in more accessible biofluids such as plasma and serum [2,3,4], which may provide insights into disease biology and progression.

Among circulating biomarkers, neurofilament light chain (NfL) is an axonal protein which has emerged as one of the most promising indicators of axonal damage [5]. NfL is a structural component of the neuronal cytoskeleton that is released into extracellular fluids following axonal injury, and is elevated across a wide spectrum of neurodegenerative disorders. Increasing evidence suggests that NfL represents a sensitive marker not only for differential diagnosis of parkinsonian syndromes [6,7,8,9], but also for monitoring the intensity of neurodegenerative processes and treatment responses [10,11,12]. Notably, elevated NfL levels precede the clinical onset of neurological disorders, including PD, with detectable levels reported up to 12–24 months prior to symptom manifestation [13,14,15,16].

Another emerging biomarker is glial fibrillary acidic protein (GFAP), an intermediate filament protein predominantly expressed by astrocytes. Increased GFAP levels are thought to reflect reactive astrogliosis and neuroinflammatory processes that accompany neurodegeneration [17]. Elevated GFAP concentrations have been reported in several neurological disorders, including PD, multiple sclerosis, frontotemporal dementia and Alzheimer disease [18,19], suggesting that astrocytic activation may represent an additional pathological mechanism contributing to disease progression. Together, NfL and GFAP provide complementary information on distinct pathological processes underlying neurodegeneration, reflecting axonal injury and astrocyte-mediated neuroinflammatory responses, respectively.

In parallel with progress regarding fluid biomarkers, neuroimaging techniques have substantially improved our ability to investigate brain alterations associated with PD. Structural magnetic resonance imaging (MRI) approaches such as voxel-based morphometry (VBM), diffusion tensor imaging (DTI), and morphometric network analyses have revealed widespread changes in white-matter (WM) microstructure, axonal integrity and interregional connectivity. These findings support the notion that PD should be considered not only as a focal dopaminergic disorder but also as a large-scale network disease affecting distributed brain systems. Within this framework, the concept of the MRI-based brain connectome has emerged as a powerful approach to model and quantify the human brain as a complex network. Graph theory provides a mathematical framework to characterize the topological organization of structural and functional brain networks, enabling the investigations of both global and regional properties of brain connectivity in healthy and pathological conditions [20,21].

By enabling the mapping of structural connections at the individual level, diffusion MRI tractography provides a non-invasive method to explore the neuroanatomical substrates underlying motor and cognitive heterogeneity observed in PD. However, conventional tractography approaches may produce a substantial number of false-positive streamlines, potentially limiting the biological interpretability of reconstructed connectivity patterns. To address this limitation, the Convex Optimization Modeling for Microstructure Informed Tractography (COMMIT) framework was developed to improve the biological plausibility of tractography reconstructions by incorporating microstructural information into the modeling of diffusion signals [22]. A subsequent extension, COMMIT2, further enhanced the specificity of reconstructed connections by introducing group-sparsity constraints aimed at suppressing spurious fiber bundles and enhancing the biological plausibility of connectome reconstruction [23].

Despite the growing interest in circulating biomarkers of neurodegeneration, their relationship with large-scale brain network organization in Parkinson’s disease remains largely unexplored. Fluid biomarkers provide valuable information about the molecular processes underlying neurodegeneration, they do not directly inform how these pathological mechanisms influence the structural organization of brain networks. Neurodegenerative processes such as axonal injury and astrocytic activation are expected to affect the integrity of WM pathways and, consequently, the topological architecture of brain connectivity.

In the present study, we investigated the relationship between circulating levels of NfL and GFAP and graph-theoretical metrics of structural brain networks derived from diffusion MRI data. Specifically, we analyzed global and nodal graph-theoretical metrics obtained from connectomes reconstructed using the microstructure-informed tractography framework COMMIT2 in a cohort of patients with PD and healthy controls (HC). By integrating blood biomarkers with advanced connectomic measures, this study aims to provide novel insights into the link between molecular markers of neurodegeneration and large-scale WM network alterations in PD.

We hypothesized that circulating biomarkers reflecting neuroaxonal damage (NfL) and astroglial activation (GFAP) would be associated with distinct patterns of alteration in the organization of structural brain networks in patients with PD.

## 2. Results

### 2.1. Patients’ Clinical Features

Cohort analysis included 73 PD patients and 34 HC. Demographic and clinical characteristics are summarized in Table 1. PD and HC subjects were matched in age (*p* = 0.21) while sex distribution differed between groups (*p* = 0.02). Montreal Cognitive assessment (MoCA) scores were significantly different between groups (*p* = 0.006). No differences were observed in NfL and GFAP levels between groups. However, significant association with MoCA scores were found only in PD patients. Specifically, within the PD cohort, higher global modularity was associated with lower MoCA score (ρ ≈ −0.25, *p* = 0.038; Figure S1). In addition, higher NfL (ρ ≈ −0.37, *p* = 0.008; Figure S2A) and GFAP levels (ρ ≈ −0.31, *p* = 0.015; Figure S2B) were associated with lower MoCA scores. Given the significant differences in sex distribution between groups, all group comparisons and correlation analyses were adjusted for age and sex.

### 2.2. Global and Local Network Differences Between HC and PD

Global network measures showed alterations in PD compared to HC. Specifically, mean strength (*p* = 0.04) and global efficiency (*p* = 0.03) were reduced in PD, whereas path length (*p* = 0.03) and modularity (*p* = 0.04) were increased. These results are summarised in Table 2.

**Figure 1:**
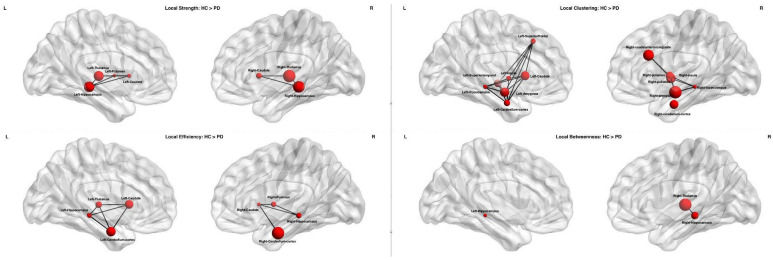
**Nodal network differences between HC and PD.** Nodes surviving FDR correction (*p*-FDR < 0.05) are displayed. From the top-left panel and proceeding clockwise, significant nodes are shown for the following network metrics: strength, clustering coefficient, betweenness centrality, and efficiency. Node size is proportional to −log10(*p*-FDR). Edges represent the normalized difference in mean structural connectivity (PD − HC) and are included for visualization purposes only. Abbreviations: PD = Parkinson’s disease; HC = healthy controls.

Nodal network analysis revealed widespread reductions in PD compared to HC. Significant decreases in local strength were observed in bilateral thalami, hippocampi and caudate nuclei, left putamen and whole brainstem. For local clustering coefficient, differences were found in bilateral cerebellar cortex, hippocampi, amygdala and insula, left superior frontal and superior temporal cortex and left caudate, right caudal anterior-cingulate, putamen and pallidum. Local efficiency was reduced in bilateral cerebellar cortex, hippocampi and caudate nuclei, as well as left thalamus and right putamen. Local betweenness centrality was decreased in bilateral hippocampi and right thalamus. All these results are summarized in Table 3 and illustrated in Figure 1.

### 2.3. Correlations Between Global or Local Network and Serum Biomarkers

In the PD subjects Spearman’s correlation analyses revealed significant associations between global network measures and serum biomarker levels after adjusting for age and sex. *p*-values were corrected for FDR.

**Figure 2:**
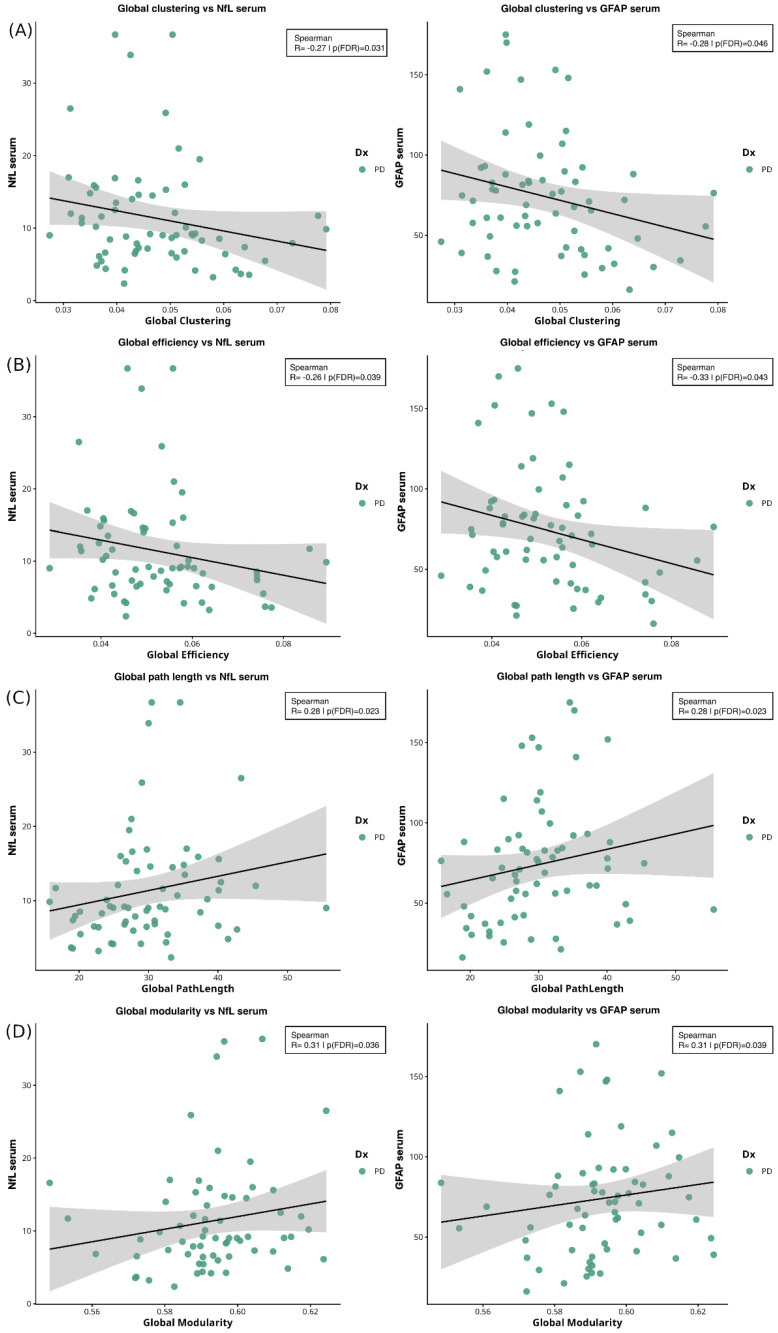
**Spearman correlations between global network measures and serum biomarkers.** (**A**) Correlation between global clustering coefficient and NfL (ρ = −0.27; *p* = 0.031) and GFAP (ρ = −0.28; *p* = 0.046) levels. (**B**) Correlations between global efficiency and NfL (ρ = −0.26; *p* = 0.039) and GFAP (ρ = −0.33; *p* = 0.043) levels. (**C**) Correlations between path length and NfL (ρ = 0.28; *p* = 0.023) and GFAP (ρ = 0.28; *p* = 0.023) levels. (**D**) Correlations between global modularity and NfL (ρ = 0.31; *p* = 0.036) and GFAP (ρ = 0.31; *p* = 0.039) levels. Shaded areas indicate 95% confidence interval of the regression line. Abbreviations: NfL = neurofilament light chain; GFAP = glial fibrillary acidic protein; PD = Parkinson’s disease.

Lower global clustering coefficient was associated (Figure 2A) with higher NfL (ρ ≈ −0.27, *p* = 0.031) and GFAP (ρ ≈ −0.28, *p* = 0.046) levels. Similarly, lower global efficiency was associated (Figure 2B) with increased NfL (ρ ≈ −0.26, *p* = 0.039) and GFAP (ρ ≈ −0.33, *p* = 0.043) levels. Conversely, higher global path length was associated (Figure 2C) with higher NfL (ρ ≈0.28, *p* = 0.023) and GFAP (ρ ≈ 0.28, *p* = 0.023) levels. Finally, higher global modularity was associated (Figure 2D) with higher NfL (ρ ≈ 0.31, *p* = 0.036) and GFAP (ρ ≈ 0.31, *p* = 0.039) levels. No significant correlations were observed in HC.

**Figure 3:**
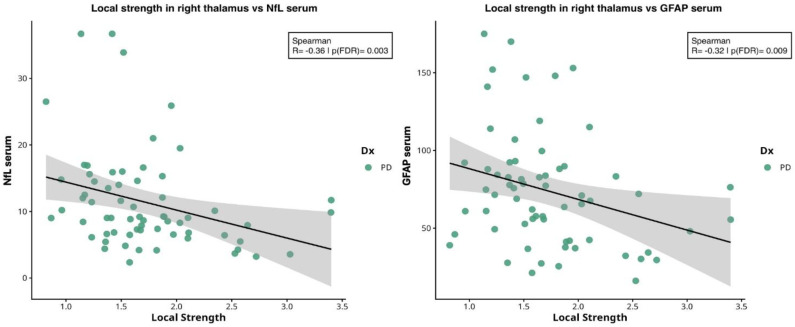
**Spearman correlations between local strength in right thalamus and serum biomarkers.** From left to right: correlation between local strength in right thalamus and NfL (ρ = −0.36, *p* = 0.003) and GFAP (ρ = −0.32; *p* = 0.009) levels. Shaded areas indicate 95% confidence interval of the regression line. Abbreviations: NfL = neurofilament light chain; GFAP = glial fibrillary acidic protein; PD = Parkinson’s disease.

We then investigated correlations between serum biomarker levels and local network measures at nodes that were significantly different between PD and HC (Table 3). Specifically, we focused on the right thalamus for local strength and betweenness, and the right cerebellar cortex for local clustering coefficient and local efficiency measures. Local strength in right thalamus was negatively correlated with NfL (ρ ≈ −0.36, *p* = 0.003) and GFAP (ρ ≈ −0.32, *p* = 0.009), see Figure 3.

**Figure 4:**
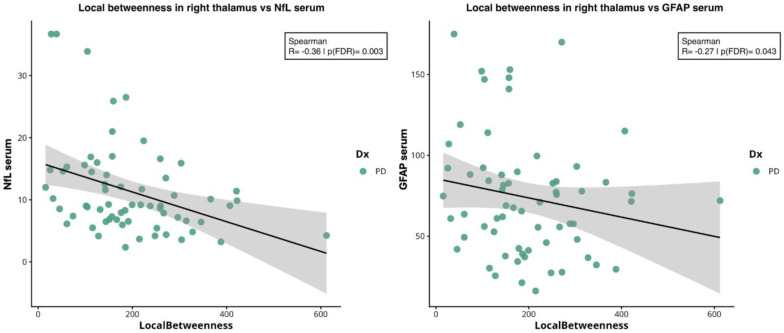
**Spearman correlations between local betweenness in right thalamus and serum biomarkers.** From left to right: correlation between local betweenness in right thalamus and NfL (ρ = −0.36; *p* = 0.003) and GFAP (ρ = −0.27; *p* = 0.043) levels. Abbreviations: NfL = neurofilament light chain; GFAP = glial fibrillary acidic protein; PD = Parkinson’s disease.

Local betweenness in right thalamus was negatively correlated with NfL (ρ ≈ −0.36, *p* = 0.003) and GFAP (ρ ≈ −0.27, *p* = 0.043), see Figure 4.

**Figure 5:**
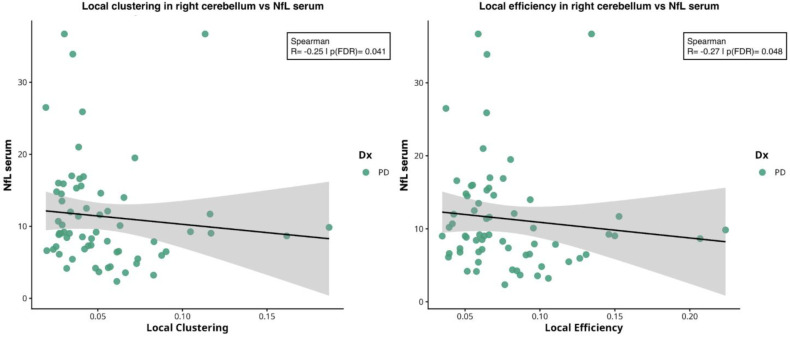
**Spearman correlations between local network measures in right cerebellum cortex and serum biomarkers.** From left to right: correlation between NfL levels with local clustering coefficient (ρ = −0.25; *p* = 0.041) and local efficiency (ρ = −0.27; *p* = 0.048) extracted in right cerebellar cortex. Abbreviations: NfL = neurofilament light chain; PD = Parkinson’s disease.

In right cerebellar cortex, local clustering coefficient (ρ ≈ −0.25, *p* = 0.041) and local efficiency (ρ ≈ −0.27, *p* = 0.048) were negatively correlated with NfL levels, see Figure 5.

Finally, additional exploratory analyses were performed to assess associations between network measures, serum biomarkers, and motor severity as measured by the MDS-UPDRS-III score. However, none of these associations reached significance.

## 3. Discussion

In this study, we investigated the relationship between circulating levels of NfL and GFAP and graph-theoretical metrics derived from diffusion MRI-based connectomes. Our findings provide evidence that alterations in diffusion-based brain connectivity in PD are associated with circulating biomarkers that reflect distinct but complementary pathological processes: axonal damage and astrocytic activation. These results support a multimodal link between large-scale network disruption and molecular blood markers.

At the global level, PD patients exhibited reduced mean strength and global efficiency, alongside increased characteristic path length and modularity, indicating a shift toward a less integrated and more segregated network organization [24]. Reduced global efficiency and increased path length suggest impaired long-range communication and a reduced capacity for efficient information transfer across the networks, whereas increased modularity reflects a tendency toward fragmentation into more isolated subnetworks [20]. Overall, these alterations support the conceptualization of PD as a large-scale structural disconnection syndrome, consistent with previous connectomic studies demonstrating widespread disruption of WM networks in PD [20,24,25].

At the regional level, PD patients showed widespread reductions in nodal network metrics across both subcortical and cortical regions. These alterations primarily involved thalamus, hippocampus, caudate and putamen, as well as cerebellar cortex and brainstem, and extended to limbic and associative cortical regions including amygdala, insula and frontal and temporal cortex. These regions are involved in circuits supporting motor control, cognitive integration and affective processing, and have been reportedly implicated in PD pathophysiology [26]. Reductions in connectivity within the putamen and thalamus are consistent with the well-established involvement of cortico-basal ganglia-thalamo-cortical circuits in PD [27,28,29,30]. Alterations involving hippocampus and cerebellum may reflect broader network dysfunction affecting cognitive processes and motor coordination. Moreover, the observed reduction in betweenness centrality within thalamic and hippocampal regions suggests a loss of hub-like properties, which may impair efficient global communication across the network. Disruption of such highly connected nodes has been proposed as a key mechanism underlying network vulnerability in neurodegenerative diseases and has been increasingly described in the PD structural connectome [26,31]. In this framework, global network disintegration may arise as a consequence of targeted vulnerability of hub regions within large-scale brain networks.

Beyond global and nodal alterations, the most relevant finding of the present study is the association between connectomic measures and circulating biomarkers. In PD patients, both NfL and GFAP levels were significantly correlated with global network properties. Higher biomarker concentrations were associated with reduced clustering coefficient and global efficiency, alongside increased path length and modularity. These results suggest that elevated biomarker levels that reflect distinct but interacting mechanisms, are linked to progressive disruption of large-scale structural connectivity [32,33,34].

NfL is a well-established marker of neuroaxonal damage, reflecting the release of cytoskeletal components following axonal injury. Since structural brain networks are fundamentally supported by WM axonal pathways, increased NfL levels can be interpreted as an index of edge-level disruption within the connectome. In this context, the observed associations between higher NfL levels and reduced global efficiency and clustering coefficient, together with increased path length and modularity, likely reflect a progressive loss of structural connections leading to impaired integration and longer communication routes across the network.

Importantly, the use of COMMIT2 filtering enhances the biological specificity of reconstructed connections by reducing false-positive streamlines and incorporating microstructural information, thereby strengthening the interpretation that NfL-related effects reflect genuine axonal loss rather than methodological artifacts. Consistent with this interpretation, previous studies have reported significant associations between circulating NfL levels and WM microstructural damage measured with diffusion MRI across several neurodegenerative conditions. For example, Spotorno and colleagues have demonstrated that increased NfL levels have been linked to reduced fractional anisotropy and widespread WM degeneration in frontotemporal dementia [35]. Similarly, longitudinal studies in multiple sclerosis have shown that higher serum NfL concentrations are associated with increased brain structural disconnectivity derived from MRI-based network mapping, supporting the role of NfL as a marker of large-scale axonal network injury [36]. Evidence from Alzheimer’s disease studies has also demonstrated correlations between plasma NfL levels and alterations in structural connectomes and diffusion-based WM metrics [34].

Together, these findings suggest that circulating NfL levels may reflect not only local axonal injury but also large-scale connectome disorganization.

In contrast, GFAP reflects astrocytic activation and neuroinflammatory processes, which may influence brain networks through mechanisms extending beyond direct structural damage. Astrocytes play a central role in maintaining synaptic and metabolic homeostasis and actively participate in neuroimmune signaling within the central nervous system. In neurodegenerative disease, astrocytes undergo reactive astrogliosis characterized by morphological and functional changes and increased expression of GFAP [37,38]. Reactive astrocytes are increasingly recognized as key modulators of disease progression, contributing to synaptic dysfunction, inflammatory signaling and structural remodeling of neural circuits across several neurodegenerative disorders [39,40]. Accordingly, elevated GFAP levels in biofluids have been associated with disease severity, brain atrophy and neurodegenerative progression [41,42]. In our cohort, the observed relationship between GFAP and increased modularity suggests a shift toward greater network segregation, potentially reflecting an adaptive or compensatory reorganization and reduced intermodular communication. Similarly, reductions in clustering coefficient and global efficiency may indicate disruption of local microcircuit organization mediated by neuroinflammatory processes. Together, these findings suggest that NfL and GFAP provide complementary but mechanistically distinct insight into network degeneration in PD. While NfL primarily reflects structural disconnection due to axonal loss, GFAP may capture astrocyte-mediated processes that contribute to network reconfiguration and functional isolation of brain regions. The convergence of these biomarkers on similar graph metrics likely reflects the interplay between degeneration and neuroinflammation in shaping large-scale network architecture [32,33].

At the regional level, nodal network metrics in the right thalamus and right cerebellum cortex were most strongly associated with biomarker concentrations. The thalamus, in particular, emerges as a critical connector hub linking molecular and network-level alterations. As a major relay structure integrating motor, cognitive, and associative information, thalamic alterations have widespread consequences on global network organization [43,44]. In our study, reduced local strength and betweenness centrality in the right thalamus were strongly associated with both NfL and GFAP levels, suggesting that this region represents a key site where neuroaxonal damage and astrocytic activation converge. This finding supports the notion of the thalamus as a biomarker-sensitive hub, whose structural integrity may reflect the combined impact of degenerative and neuroinflammatory processes [43,45]. Reduced nodal strength and betweenness in the thalamus, together with alterations observed in other basal ganglia structures, are consistent with the central role of cortico–basal ganglia circuits in PD [46,47].

Disruption of thalamic connectivity has been widely reported in PD and is thought to contribute to both motor and non-motor symptoms through altered thalamo-cortical communication [46,47].

The cerebellum has also been increasingly implicated in PD pathophysiology, both as part of compensatory mechanisms and as a contributor to network reorganization associated with motor and cognitive dysfunction [29,48]. In our study, local clustering coefficient and efficiency in the right cerebellar cortex were associated with NfL but not with GFAP levels. This finding suggests that cerebellar network alterations may be more directly related to microstructural integrity and axonal damage, consistent with the known sensitivity of diffusion MRI metrics to WM disruption, particularly in relation to axonal organization and myelin integrity [49]. In contrast, the absence of significant GFAP association in this region may indicate a lesser contribution of astrocyte-mediated inflammatory processes, highlighting regional heterogeneity in the mechanisms underlying network alterations.

From a network perspective, astrocytic activation may also contribute to the vulnerability of specific brain circuits to neurodegenerative processes. According to network-based models of disease propagation, pathological processes may spread along anatomically connected pathways, progressively disrupting large-scale brain networks [50,51]. Astrocytes play a critical role in regulating synaptic activity, metabolic support, and neuroinflammatory responses within these networks, and astrocyte-mediated inflammatory signaling may further exacerbate the propagation of pathology across interconnected regions, contributing to large-scale network disruption. In our study, we did not observe significant differences in NfL and GFAP levels between PD patients and HC. This is consistent with previous evidence showing that NfL levels in PD often exhibit substantial overlap with healthy individuals, particularly in cohorts with moderate disease severity, and are more closely related to disease heterogeneity and progression than to case–control diagnostic separation [6,9,10]. In this context, the observed associations with network measures suggest that these biomarkers may capture individual variability in neurodegenerative burden rather than group-level differences.

Overall, the integration of connectomic metrics with circulating biomarkers provides complementary insights into the mechanisms underlying neurodegeneration in PD. Diffusion MRI captures the structural architecture of WM pathways, while blood-based biomarkers reflect molecular indicators of neuronal injury and astrocytic activation. Combining these approaches offers a multimodal framework to monitor disease-related network changes, potentially supporting early diagnosis and the identification of therapeutic targets.

Limitations include the cross-sectional design, the indirect nature of diffusion MRI metrics that may be influenced by technical and modeling constraints, and the relatively small HC sample size, which may affect generalizability.

## 4. Materials and Methods

### 4.1. Subjects

The study enrolled 73 subjects with PD and 34 HC. Patients were recruited consecutively between 2021 and 2024 at the Neurology Institute and Neuroscience Research Centre of the University of Catanzaro, Italy. PD diagnosis was made by movement disorder specialists according to the MDS international diagnostic criteria [1]. All patients underwent a neurological examination in the “OFF” state (off medications overnight). Clinical data were collected, including the MDS-Unified Parkinson’s Disease Rating Scale (MDS-UPDRS) scale [52], MoCA and the Mini-Mental State Examination (MMSE) [53]. MoCA scores were interpreted according to normative data validated for the Italian population. Specifically, studies in Italian cohorts have identified an adjusted cut-off of >17.54 [54,55]. Based on these studies, an average score of 20, as observed in HC, lies in the normal performance range, while the mean value for PD patients can be considered slightly above a borderline performance. MoCA was used as a screening tool rather than a standalone diagnostic measure of cognitive status. Additionally, all patients underwent a 3 T brain MRI scan with a recently described protocol [56] to rule out secondary causes of Parkinsonism. Exclusion criteria for PD patients were: clinical features suggestive of other diseases and MRI abnormalities such as neoplasms, lacunar infarctions in the basal ganglia, or diffuse subcortical vascular lesions. Thirty-four age matched controls were also recruited for this study. HC were recruited from a cohort of volunteers who participated in one of the MRI programs at our center, and they were selected based on the following inclusion criteria: absence of clinically manifest cognitive decline, as well as no history of stroke, neurological, or psychiatric disorders.

The study was approved by the local ethics committee of the University Magna Graecia of Catanzaro, Italy, and all participants provided written informed consent in accordance with the Declaration of Helsinki.

### 4.2. MRI Protocol and Image Analysis

All subjects underwent 3 T MRI scanning (Biograph mMR, Siemens Healthineers, Forchheim, Germany) using a 16-channel PET-transparent head/neck coil. The multimodal protocol included both conventional and research MR sequences. For each participant enrolled, the following sequences were analyzed: (i) whole-brain T1 weighted (MPRAGE, 176 sagittal planes, 256 × 256 mm^2^ field of view, voxel size 1 × 1 × 1 mm^3^, TR/TE/TI = 2300/2.34/900 ms, flip angle 8°, TA = 5′12″); (ii) echo-planar imaging diffusion-weighted scans (EPI, 70 slices, phase encoding A>>P, 250 × 250 mm^2^ field of view, voxel size 2.5 × 2.5 × 2.3 mm^3^, TR/TE = 8900/88 ms, b = 1000 s/mm^2^, diffusion weighting along 64 gradient directions, number of b0 images = 9, TA = 11′36″).

Structural images were processed automatically using standard freesurfer pipeline (https://surfer.nmr.mgh.harvard.edu/, version 7.4.2, accessed on 10 March 2026) with the recon-all script, as described previously [57]. From this pipeline, the Desikan-Killiany cortical and subcortical parcellation atlas was obtained for each subject [58].

Diffusion-weighted imaging (DWI) data were processed using the TractoFlow pipeline (version 2.4.4) [59], a fully automated and reproducible workflow based on Nextflow and Singularity containers, which handles the entire processing from raw images to tractogram generation and Diffusion Tensor Imaging (DTI) metric computation (https://tractoflow-documentation.readthedocs.io/en/latest/, accessed on 10 January 2026). PInstead of adopting the standard TractoFlow pipeline for T1-weighted images, the previously computed FreeSurfer outputs were used, including cortical and subcortical segmentations and surfaces to guide registration, seeding and tractography.

DWI images were processed through standard steps including denoising with MRtrix (version 3.0.8), brain extraction with FSL BET (version 6.0.7.18), correction on eddy currents and geometric distortions with FSL Topup/Eddy, bias field correction with ANTs (version 2.6.2) and isotropic resampling with Dipy. Whole-brain tractography was generated using anatomically constrained tractography (ACT) as implemented in MRtrix3, with streamlines seeded at the gray matter–white matter interface. Anatomical priors were derived from the Freesurfer Desikan–Killiany atlas, comprising 84 cortical and subcortical regions, and were used to guide streamline propagation and filtering rather than to directly constrain seeding to specific regions. The brainstem was further segmented into substructures [60], resulting in a total of 85 nodes for each subject. This approach is necessary to perform anatomically constrained tractography. To improve anatomical plausibility, the tractogram was filtered using COMMIT2 [23], which assigns a weight to each streamline based on its contribution to the measured DWI signal, effectively reducing false-positive connections. The filtered tractogram was used to compute the structural connectome using tck2connectome (MRtrix3), generating a subject-specific weighted adjacency matrix in which nodes corresponded to 85 cortical and subcortical regions defined by the Desikan-Killiany atlas (including the brainstem). Edges were defined as the sum of COMMIT2-derived streamline weights connecting each pair of regions of interest, rather than simple streamline counts. Specifically, each streamline was assigned a weight reflecting its contribution to the diffusion signal as estimated by COMMIT2, and edge weights were computed by summing these contributions across all streamlines linking a given pair of regions. This approach ensures that the resulting weighted connectome reflects both the anatomical segmentation of the subject and microstructure-informed tractography filtering, providing a more biologically meaningful estimate of structural connectivity.

### 4.3. Network Measures

Graph theoretical measures were computed for each subject using the Brain Connectivity Toolbox (BCT) [61] implemented in MATLAB environment (version R2025b). Individual connectivity matrices were proportionally thresholded at a fixed network density of 0.15 in order to ensure comparable network sparsity across subjects and to reduce potential biases related to inter-individual differences in overall connectivity strength [61,62]. At the global level, the following metrics were extracted: mean global strength, clustering coefficient, global efficiency, characteristic path length and modularity. At the nodal level, the following local metrics were computed: betweenness centrality, local clustering coefficient, local efficiency and nodal strength.

### 4.4. Serum Biomarker Assessment

For each subject, serum was collected between 9 a.m. and 12 p.m. in BD Vacutainer™ SST™ Serum Separation Tubes (BD, Franklin Lakes, NJ, USA) and processed within 30 min by centrifugation at 3000 rpm at 4 °C for 10 min, aliquoted and stored at −80 °C until use. For biomarkers assessment, aliquots were thawed overnight at 4 °C, mixed thoroughly and centrifuged at 2200 rpm for 15 min. Ultrasensitive single molecule array (SIMOA) on a fully automated Quanterix HD-X™ Automated Immunoassay Analyzer (Quanterix, Billerica, MA, USA) was employed to assess all the biomarkers of interest. Specifically, Nf-L and GFAP were collectively evaluated by using the Neurology 4-Plex E Advantage PLUS kit (104465, Quanterix). All the measurements were performed in duplicates following manufacturer’s instructions blinded to the patients’ diagnoses [5].

### 4.5. Statistical Analysis

Statistical analysis was performed using RStudio (Version 2023.06.1). The normality of data distribution was assessed using the Shapiro-Wilk test. Differences between HC and PD subjects were evaluated using analysis of covariance (ANCOVA), with group as independent variable and age and sex included as covariates to control for their potential confounding effects. *p*-values were adjusted for multiple comparisons using Benjamini–Hochberg false discovery rate (FDR) correction, and statistical significance was set at *p* < 0.05. Global and nodal network metrics were first compared between PD and HC subjects. For global network metrics, FDR correction was applied across all global measures (mean strength, clustering coefficient, global efficiency, path length and modularity). For nodal network metrics, FDR correction was applied across all brain regions separately for each metric.

Association between biomarker levels and graph-theoretical metrics were assessed using partial correlation analyses controlling for age and sex. Spearman partial correlations were computed due to non-normal data distribution, focusing on global and nodal network metrics that showed significant group differences.

Exploratory analyses were also conducted to assess associations between network measures, serum biomarkers, and motor severity (MDS-UPDRS-III score), using partial Spearman correlations controlling for age and sex, with FDR correction.

## 5. Conclusions

In conclusion, this study provides novel evidence linking circulating biomarkers of neurodegeneration to large-scale structural brain network alterations in PD. Our findings support a model in which neuroaxonal damage and astrocytic activation contribute through distinct but interacting mechanisms to the disruption and reorganization of brain connectivity. Integrating blood-based biomarkers with advanced connectomic approaches may offer a powerful strategy to better characterize disease mechanisms, identify vulnerable network nodes, and develop multimodal markers of neurodegeneration.

## Figures and Tables

**Table 1:** Demographic and clinical data.

Data	PD (n = 73)	HC (n = 34)	*p*-Value
Sex (M/F)	24/49	14/20	**0.01 ^a^**
Age at examination, years	64.6 (8.53) ^b^	62.4 (8.3) ^b^	0.21 ^c^
Disease duration, years	5.0 (6.0) ^d^	/	/
MDS-UPDRS-III score	30.0 (22.0) ^d^	/	/
MoCA	17.7 (4.0) ^b^	20.3 (4.1) ^b^	**0.006 ^c^**
SIMOA NfL 4plex serum (pg/mL)	9.1 (7.9) ^d^	7.8 (4.4) ^d^	0.10 ^e^
SIMOA GFAP 4plex serum (pg/mL)	71.0 (42.1) ^d^	66.5 (36.5) ^d^	0.91 ^e^

Demographics and clinical data of study participants. Abbreviations: PD = Parkinson’s disease subjects; HC = healthy controls; MDS-UPDRS-III = Movement Disorder Society—Unified Parkinson’s Disease Rating Scale-part III (Motor Examination); MoCA = Montreal Cognitive Assessment; NfL = neurofilament light chain; GFAP = glial fibrillary acidic protein. Significant *p* values (*p* < 0.05) are highlighted in bold. Normal distribution of data was tested with the Shapiro-Wilk test. ^a^ Fisher’s exact test. ^b^ Data are expressed as mean (standard deviation). ^c^ *t*-test. ^d^ Data are expressed as median (IQR). ^e^ Mann-Whitney U test.

**Table 2:** Global Metric values between HC and PD.

	HC	PD	*p*-FDR
Mean strength	1.26 (0.29)	1.17 (0.26)	**0.04**
Clustering coefficient	0.05 (0.01)	0.04 (0.01)	0.09
Global efficiency	0.06 (0.01)	0.05 (0.01)	**0.03**
Path length	26.94 (6.93)	30.20 (7.70)	**0.03**
Modularity	0.57 (0.02)	0.59 (0.02)	**0.04**

Data are expressed as mean (standard deviation). Significant *p* values (*p* < 0.05) are highlighted in bold. All *p*-values are corrected for False Discovery Rate (FDR).

**Table 3:** Nodal Metric values between HC and PD.

	ROI	HC	PD	*p*-FDR
*Local Strength*	Left Thalamus	2.03 (0.64)	1.72 (0.57)	0.009
	Right Thalamus	2.09 (0.72)	1.71 (0.57)	0.005
	Left Hippocampus	0.95 (0.33)	0.79 (0.29)	0.008
	Right Hippocampus	0.94 (0.27)	0.78 (0.28)	0.006
	Left Caudate	2.69 (1.18)	2.23 (1.04)	0.04
	Right Caudate	2.81 (1.18)	2.29 (1.06)	0.03
	Left Putamen	1.99 (0.66)	1.74 (0.61)	0.04
	Brainstem	1.26 (0.30)	1.10 (0.33)	0.008
*Local Clustering coefficient*	Left Cerebellar Cortex	0.09 (0.06)	0.06 (0.04)	0.02
	Right Cerebellar Cortex	0.09 (0.07)	0.05 (0.03)	0.01
	Left Hippocampus	0.03 (0.008)	0.02 (0.007)	0.04
	Right Hippocampus	0.02 (0.005)	0.01 (0.003)	0.04
	Left Amygdala	0.03 (0.01)	0.03 (0.01)	0.01
	Right Amygdala	0.03 (0.01)	0.02 (0.008)	0.004
	Left Insula	0.03 (0.01)	0.02 (0.008)	0.03
	Right Insula	0.04 (0.01)	0.03 (0.008)	0.03
	Left Superior Frontal	0.04 (0.01)	0.03 (0.008)	0.03
	Left Superior Temporal	0.04 (0.01)	0.03 (0.01)	0.01
	Left Caudate	0.06 (0.02)	0.05 (0.01)	0.01
	Right caudal anterior cingulate	0.05 (0.02)	0.04 (0.02)	0.005
	Right Putamen	0.04 (0.01)	0.03 (0.01)	0.01
	Right Pallidum	0.04 (0.03)	0.02 (0.01)	0.03
*Local Efficiency*	Left Cerebellar Cortex	0.13 (0.08)	0.08 (0.05)	0.01
	Right Cerebellar Cortex	0.12 (0.08)	0.07 (0.02)	0.005
	Left Caudate	0.10 (0.03)	0.08 (0.03)	0.01
	Right Caudate	0.09 (0.03)	0.08 (0.02)	0.03
	Left Hippocampus	0.05 (0.01)	0.04 (0.01)	0.01
	Right Hippocampus	0.05 (0.01)	0.04 (0.01)	0.01
	Left Thalamus	0.07 (0.02)	0.06 (0.02)	0.02
	Right Putamen	0.08 (0.02)	0.06 (0.02)	0.02
*Local Betweenness*	Left Hippocampus	147.49 (70.63)	107.85 (79.48)	0.007
	Right Hippocampus	109.44 (152.53)	79.19 (61.12)	0.004
	Right Thalamus	285.08 (150.33)	192.41 (111.60)	0.001

Data are expressed as mean (standard deviation). All *p*-values are corrected for False Discovery Rate (FDR).

## Data Availability

The data presented in this study are available on reasonable request from the corresponding author due to privacy restrictions.

## Supplementary Materials

The following supporting information can be downloaded at: https://www.mdpi.com/article/10.3390/ijms27093934/s1.

## Abbreviations

The following abbreviations are used in this manuscript:

PD	Parkinson’s disease
HC	Healthy controls
NfL	Neurofilament light chain
GFAP	Glial fibrillary acid protein
DTI	Diffusion tensor imaging
WM	White matter
COMMIT	Convex Optimization Modeling for Microstructure Informed Tractography
BCT	Brain connectivity toolbox
FDR	False discovery rate
MoCA	Montreal Cognitive assessment
ACT	Anatomically constrained tractography
