# Supplementary material for: Serum Neurofilament Light Chain and GFAP Levels Are Associated with Structural Brain Connectivity in Parkinson’s Disease
Source: Int J Mol Sci. 2026 Apr 28;27(9):3934. doi: 10.3390/ijms27093934 (PMC13163883; doi:10.3390/ijms27093934)
Supplement: Supplementary file 1 [file ijms-27-03934-s001.zip › ijms-4266723-Supplementary_Materials.pdf]

Supplementary figures

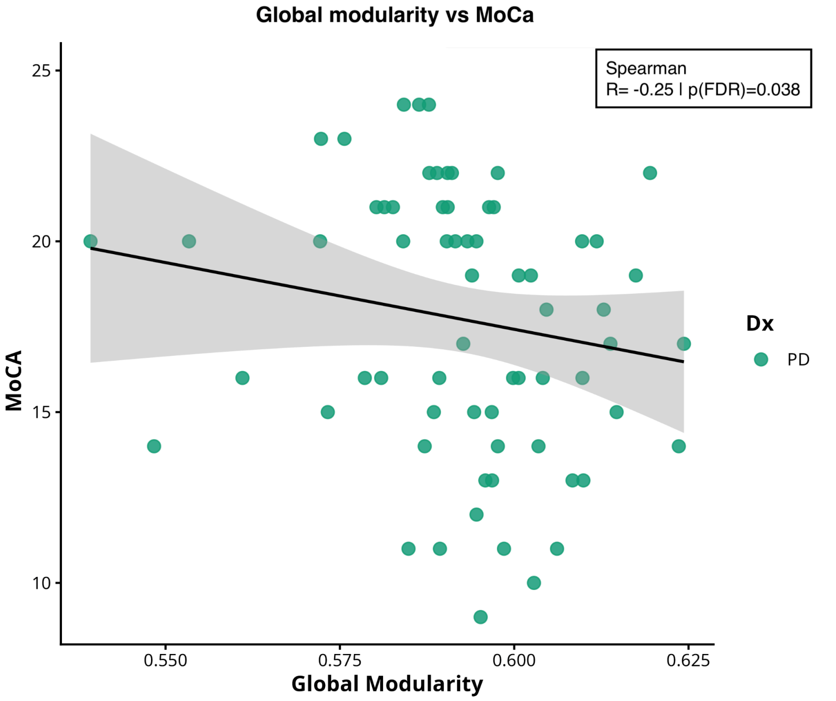

Supplementary Figure S1: Correlations between global network measures and clinical scores. Correlations between global modularity and MoCA score (ϱ = -0.25; p = 0.038). Abbreviations: MoCA = Montreal Cognitive Assessment; PD = Parkinson's disease.

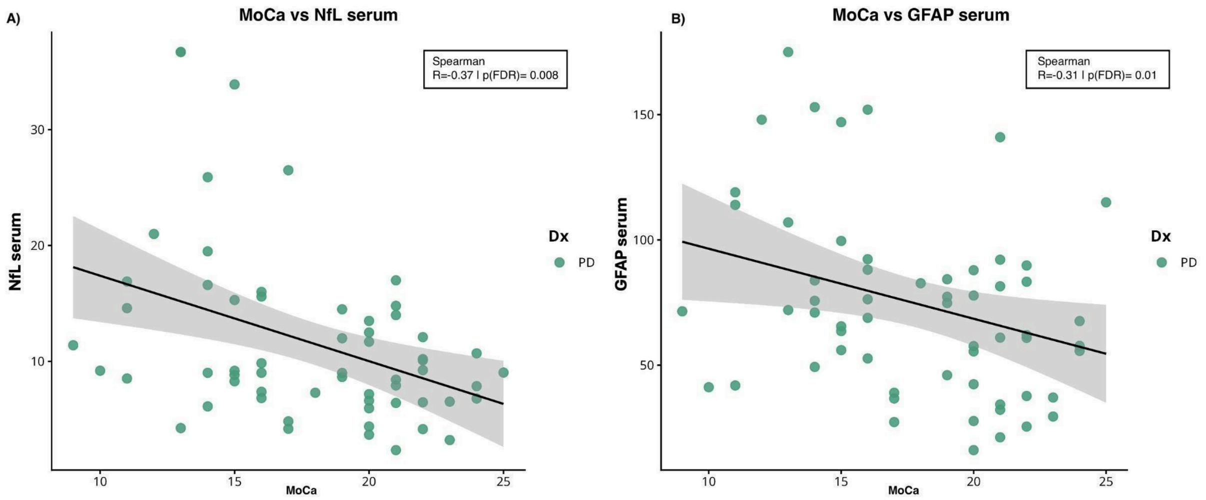

Supplementary Figure S2: Correlations between clinical scores and serum biomarkers. (A) Correlations between NfL levels and MoCA score (ϱ = -0.27; p = 0.031). (B) Correlations between GFAP levels and MoCA score(ϱ = -0.33; p = 0.043). Abbreviations: MoCA = Montreal Cognitive Assessment; NfL = neurofilament light chain; GFAP = glial fibrillary acidic protein; PD = Parkinson's disease.
